# In Search of Outliers. Mining for Protein Kinase Inhibitors Based on Their Anti-Proliferative NCI-60 Cell Lines Profile

**DOI:** 10.3390/molecules25081766

**Published:** 2020-04-11

**Authors:** George Nicolae Daniel Ion, George Mihai Nitulescu

**Affiliations:** Faculty of Pharmacy, “Carol Davila” University of Medicine and Pharmacy, Traian Vuia 6, 020956 Bucharest, Romania; daniel.ion@drd.umfcd.ro

**Keywords:** protein kinase inhibitors, anti-proliferative fingerprint, anticancer drug screening, data-mining, NCI-60 cells, drug discovery, targeted therapy, drug repurposing

## Abstract

Protein kinases play a pivotal role in signal transduction, protein synthesis, cell growth and proliferation. Their deregulation represents the basis of pathogenesis for numerous diseases such as cancer and pathologies with cardiovascular, nervous and inflammatory components. Protein kinases are an important target in the pharmaceutical industry, with 48 protein kinase inhibitors (PKI) already approved on the market as treatments for different afflictions including several types of cancer. The present work focuses on facilitating the identification of new PKIs with antitumoral potential through the use of data-mining and basic statistics. The National Cancer Institute (NCI) granted access to the results of numerous previously tested compounds on 60 tumoral cell lines (NCI-60 panel). Our approach involved analyzing the NCI database to identify compounds that presented similar growth inhibition (GI) profiles to that of existing PKIs, but different from approved oncologic drugs with other mechanisms of action, using descriptive statistics and statistical outliers. Starting from 34,000 compounds present in the database, we filtered 400 which displayed selective inhibition on certain cancer cell lines similar to that of several already-approved PKIs.

## 1. Introduction

Protein phosphorylation is a reversible process that consists of the addition of a phosphate group to the hydroxyl group of serine, threonine or tyrosine residues of protein substrates and occurs through protein kinases [1]. It is one of the most important cellular mechanisms of regulation in signal transduction, protein synthesis, cell growth and proliferation, thus, the deregulation of different kinases is implicated in the pathogenesis of numerous diseases with inflammatory, nervous or cardiovascular components [2,3]. Protein kinases’ impact in humans has transformed them into one of the most “hunted” drug targets in the pharmaceutical industry in the past years, with almost one third of pharmacological targeting in drug discovery being directed towards protein kinase inhibition [4].

Protein kinase inhibitors (PKI) can be classified based upon the structures of their drug-enzyme complexes. Type I, I½ and II inhibitors bind in the adenine pocket-forming hydrogen bonds with the hinge region of the protein kinase, while type III and IV are allosteric inhibitors, and type V are bivalent inhibitors binding to two different regions of the enzyme. The type VI compounds are irreversible inhibitors covalently binding to the enzyme [5,6]. Analyses of the chemical structure profile of PKI indicated that their chemical space is narrower, tend to have a rod-like or a rod-disc shape, and share a group of common chemical scaffolds [7,8].

Due to their involvement in the regulation of processes like signal transduction, protein synthesis, cell growth and proliferation, protein kinases play an important role in tumoral development in particular, as in most cancers the functions of various protein kinases are reported to be deregulated [1,9]. A great number of small molecules have been developed in the last decades to specifically or selectively target protein kinases as antitumor therapies [1,10,11]. Imatinib was the first kinase inhibitor to reach the market in 2001, and soon became the first-line therapy for chronic myelogenous leukemia patients [12,13]. Second generation PKIs (dasatinib, nilotinib, bosutinib) and third-generation PKIs (ponatinib) were rationally designed to target with high specificity the imatinib-resistant forms of Bcr-Abl oncoprotein [12,14].

Activating mutations of the epidermal growth factor receptor (EGFR) were identified as the major oncogenic driver of non-small cell lung cancers (NSCLC) and therefore considered an attractive target for drug development [15]. Gefitinib and erlotinib were the first two reversible inhibitors of EGFR, followed by the second generation of inhibitors (afatinib, dacomitinib, neratinib and canertinib) designed to overcome clinical resistance [16]. The third generation of EGFR inhibitors (osimertinib) was developed to target the resistance produced by the T790M mutation [17], where the aberrant change of the normal threonine with a methionine as the gatekeeper residue increases the affinity for ATP [18]. Close to 60% of patients with melanoma harbor various mutations of B-RAF that cause activation of the MAPK pathway. Vemurafenib and dabrafenib were developed to target the BRAFV600E mutation and were approved for advanced-stage melanoma treatment [19,20]. Several vascular endothelial growth factor receptor (VEGFR) inhibitors have been developed as anti-angiogenic agents [21]. Overall, the U.S. Food and Drug Administration (FDA) has approved a number of small-molecule protein kinase inhibitors for the treatment of malignant diseases. The most frequent targets of these drugs are Bcr-Abl, B-Raf, VEGFR, EGFR and anaplastic lymphoma kinase (ALK) [22].

Discovery and development of new anticancer clinical candidates is a major direction of the pharmaceutical industry, as well as government and non-government organizations [23]. Since its establishment in 1955 by the U.S. National Cancer Institute (NCI), the Cancer Chemotherapy National Service Center (CCNSC) systematic screening program has had a major impact on advancing cancer therapies and significantly changed the traditional drug discovery process [24,25]. The NCI continuously improved the screening systems and in the 1990s a panel of 60 human cancer cell lines was established, representing nine major tissue types (brain, blood and bone marrow, breast, colon, kidney, lung, ovary, prostate and skin). Over the last decades, compounds submitted by investigators have been screened against this NCI-60 panel to determine their growth inhibition effect [26,27,28].

A compound is first tested at a single concentration and then, if found active, it is tested at five different concentrations with 48 h drug exposure, and 50% growth inhibition (GI50), total growth inhibition (TGI) and 50% lethal concentration (LC50) are computed [26]. Data analysis tools such as the NCI’s Developmental Therapeutics Program (DTP)’s COMPARE algorithm use these outputs on all 60 cancer cell lines to create a fingerprint profile that allows classification and can predict the mechanism of action [29,30]. The fingerprint of cellular response in the NCI-60 assay can be used to determine similar prototype compounds, and the usefulness of this data-mining approach has been demonstrated in various studies [31,32,33,34].

The objective of this research was to find a simple, yet powerful method to identify a correlation between the NCI-60 anti-proliferative fingerprint and the pharmacological mechanism in order to search for new PKI independent of chemical scaffolds.

For this purpose, we propose generating a database for predicting the inhibition potential of protein kinases based on the growth inhibition profile provided by the NCI’s DTP (Developmental Therapeutics Program) project for a large number of compounds. The vast amount of data to be analyzed would make a discriminant analysis too complex to be computed by accessible computers and data-processing tools for all of the compounds provided by the NCI. Additionally, the lack of results for a large number of cell lines, on which some compounds haven’t been tested on, would render thousands of compounds not available for several types of analyses, as even one missing result out of a series of 60 can determine a compound not to be taken into account in some statistical approaches (for example, Pearson correlation or linear regression analyses).

The current study aims to make use of some of the publicly available, free data provided by the NCI, as in vitro tests have already been made for these compounds. This data, if correctly interpreted, could help drug discovery chemists to identify probable overlooked, druggable compounds or promising scaffolds suitable for inhibiting a specific target which may have been formerly avoided, either accidentally or due to insufficient information.

This work describes the creation of a prediction dataset containing NCI-60 anti-proliferative fingerprints of PKIs and non-PKI approved drugs and the application of its predictive potential on a testing set extracted from NCI-provided data, in order to identify structures suitable for PKI development.

## 2. Results

### 2.1. Testing Set Creation

NCI database interrogation for the creation of the testing set was performed through several data preparation and cleaning steps, described in the Materials and Methods section. Filtering in vitro test results expressed only as GI50 concentrations in μM units formed a starting set of 34,583 compound lines, from which only 16,240 compounds displayed at least one statistical outlier, either upper or lower. A selection for compounds with at least 50 shown test results out of 60 totally possible concluded in a final testing set containing 9137 compounds, the others being discarded due to insufficient data. The in vitro GI50 results were transformed to the corresponding negative log values (pGI50), for easier data management, and the whole set contained 33,861 missing datapoints, representing only 6.18% of total possible results. Descriptive statistics for the testing set are presented in Table 1.

### 2.2. Predictive Set Creation

Generation of the predictive set was realized in two stages, resulting in two groups of compounds—one of PKIs and one of other approved oncologic drugs (AODs). Out of 218 PKIs extracted from the Protein Kinase Inhibitor Database (PKIDB), only 21 were traceable by chemical or numerical identifiers and have been tested and described in the NCI database. After the selection of compounds presenting no more than 10 missing pGI50 values out of 60 totally possible, only 18 compounds remained, representing the PKI group. The approved oncologic drugs set (AOD) was formed similarly, and after the same data preparation, out of 120 extracted compounds, 80 AODs remained. The resulted predictive set, formed of 18 PKI and 80 AOD compounds tested on the NCI-60 panel, contained altogether 171 missing datapoints, which had to be replaced with average values of the other results in each compound line. A statistical description of the predictive set can also be found in Table 1.

### 2.3. Predictive Score Design

#### 2.3.1. Cell Line Weight Factors

Cell line weight factors, further referred to as “weights” (details in Section 4.3.1), were generated for each cell line for the predictive set, based on the frequencies of outliers in each group, PKI and AOD, and are presented in Table 2, along with cell lines origin. The formula for weights calculation is presented in the Methods and Materials section. Positive weights are attributed to the cell lines that presented more outliers in the PKI group than the AOD group, representing cell lines that are more sensitive or more resistant to a compound with protein kinase inhibition mechanism.

Idealizing for simplification, a high weight factor for a certain cell line, close to 100, implies that an outlier value for that line will most of the time appear for one of the compounds in the PKI group rather than for one in the AOD group, and therefore inhibiting the proliferation of that specific cell line is characteristic to PKIs. A weight factor of 0 indicates equal probabilities of appearance in both of the groups, therefore the respective cell line is not significant for identifying a potential PKI.

#### 2.3.2. Predictive Score Calculation

A score was generated based on cell line weights (explained in Section 4.3.2) and the quality of one datapoint of being an outlier in the corresponding NCI-60 array. This score roughly estimates the potential of a compound being a PKI, the higher values indicating an increased probability of inhibiting a protein kinase, and values lower than 10 indicating a reduced likelihood of the compound being a kinase inhibitor. Scores for the 9137 testing set compounds ranged from −74.58 to 118.33, with an average of −1.25, a median of −1.94 and SD of 18.76.

#### 2.3.3. Data-Mining Results Analysis

Out of 9137 compounds of the testing set, 409 resulted in displaying score values above 10 while also presenting upper fence values larger than 6, these compounds appearing to be the most promising potential PKIs. Data regarding the selected potential PKIs can be found in Supplementary material (Table S4), while descriptive statistics regarding these compounds are presented in Table 3.

The 409 potential PKIs resulting from the prediction were analyzed using chemical database manager DataWarrior 5.2.1 software [35]. Based on their chemical structures, several physicochemical descriptors were generated, along with their calculated descriptive statistics. The range and standard deviation values of these descriptors depict a very heterogeneous dataset, as can be observed in Table 4.

Bemis–Murcko skeletons represent chemical frameworks with only the rings and the linker atoms connecting them [36,37]. They were generated in order to assess the diversity of the chemical space represented by the predicted PKIs molecules [38]. This analysis resulted in 222 different skeletons describing the whole set, meaning that, on average, one molecular skeleton described less than two of the predicted potential PKIs molecules. Structures have little in common, as the most encountered skeleton was that described by the hexagon graph, which represented only 26 compounds, meaning probably the only common element for these compounds was containing a six-membered cycle in their molecule.

The large number of compound skeletons, along with the high range values for all of the descriptors computed, imply that the 409 compounds are structurally very diverse. Additionally, when corroborating the skeletons with the descriptives of molecular weight and number of atoms, it is readily understandable that these compounds come from all classes of molecules, ranging from small molecules to organometallic complexes to polypeptides and macrocyclic structures.

**Figure 1:**
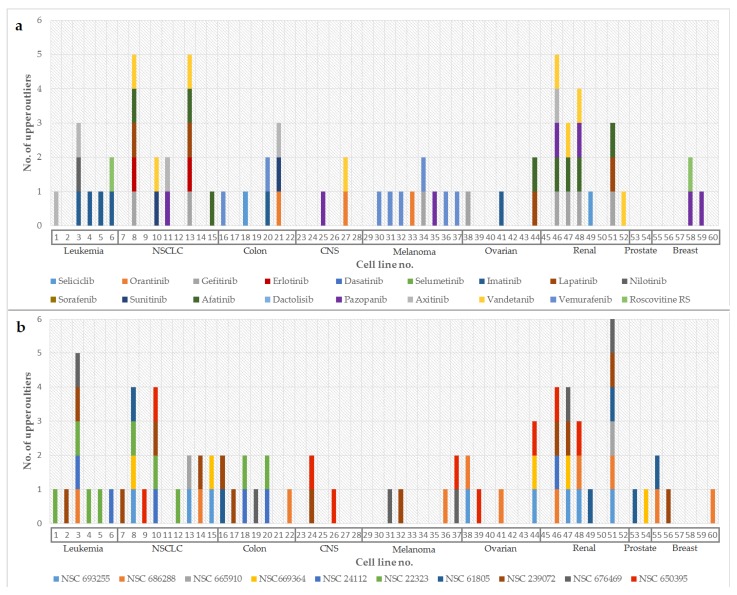
(**a**) Distribution of the pGI50 upper outlier values obtained for the NCI-60 cancer cell lines for the 18 PKIs from the predictive set. (**b**) Distribution of the pGI50 upper outlier values obtained for the NCI-60 cancer cell lines for the top 10 best-scoring compounds illustrated in Table 5. Cell line numbers correspond to cell line names as initially presented in Table 2 and are separated by tissue types for easier interpretation. The graph shows colored bars corresponding to a certain compound wherever the pGI50 value was identified as being an upper outlier for that compound. By comparison, some similarities between profiles of some of the PKIs from the predictive set and the top 10 predicted potential PKIs can be observed.

The top 10 best-scoring compounds, identifiable by NSC (Cancer Chemotherapy National Service Center number) are presented in Table 5. Similarities between a known PKI from the predictive set and some of the potential PKIs predicted by the developed method can be observed in Figure 1, where the distributions of upper outliers for both sets (18 known PKIs and top 10 potential PKIs) are presented. For example, out of 8 outliers for each of the compounds, 5 cell lines (8, 13, 15, 47 and 48) can be seen to be common outliers for both afatinib (dark green in Figure 1a) and NSC 693255, also known as tyrphostin AG-1478 (light blue in Figure 1b).

**Figure 2:**
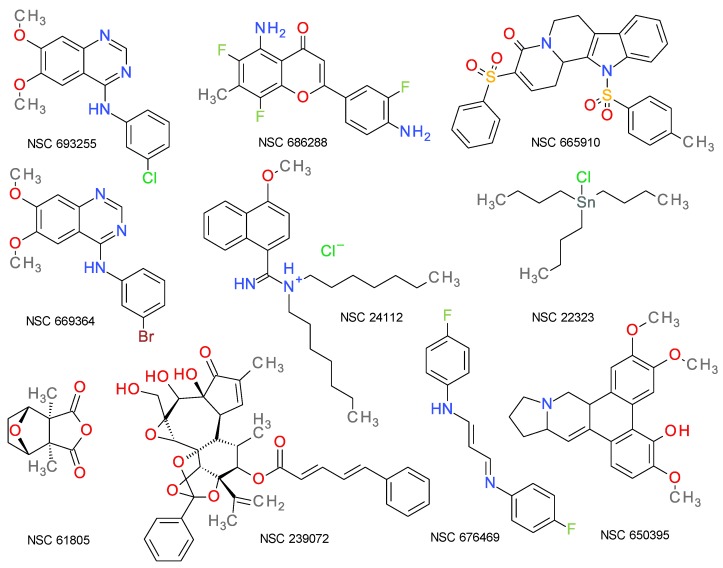
Chemical structures of the top 10 best-scoring compounds based on the prediction algorithm.

The majority of the chemical structures of the top 10 best-scoring compounds predicted to target protein kinases don’t share the typical structure of most PKIs, supporting thus the utility of this method to help the medicinal chemist to find new leads for the design of future PKIs. The structures of these compounds are presented in Figure 2.

#### 2.3.4. Predictive Method Validation

**Figure 3:**
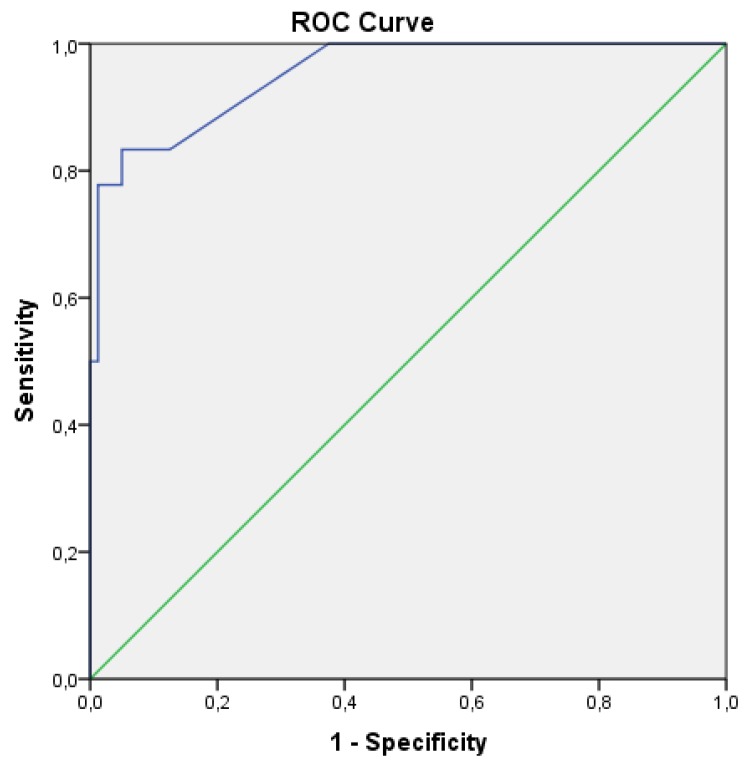
Receiver operating characteristic (ROC) analysis diagram. Maximum area under the curve = 0.952, with standard error = 0.025.

Internal validation of the score results was performed in the form of a ROC (receiver operating characteristic) curve analysis, assessing the accuracy of the scoring system on the predictive set. The ROC curve graph is presented in Figure 3. Descriptives for the scores of the predictive set compounds can be found in the Supplementary Material section, Table S3. Based on this analysis, the cut-off value for the score was set to be 10.21, with an area under the curve of 0.952, sensitivity of 0.833 and 1–specificity value of 0.50.

When analyzing the scores for the compounds of the predictive set, it was observed that 15 PKIs out of 18 forming the PKI group presented scores higher than the cut-off value (with NSC 732517, NSC 741078 and NSC 747971 obtaining score values of 0, representing false-negative results), while from the AOD group, 4 out of 80 compounds scored higher than 10.21, (NSC 26271, NSC 138783, NSC 719345 and NSC 719627, with score values of 37.50, 17.50, 17.64 and 18.89, respectively), representing false-positives. Therefore, judging by the cut-off values, the scoring method has a calculated precision rate of 0.789 and a recall rate of 0.833.

Additionally, external validation of the method was performed using NCI’s COMPARE algorithm, by correlating each of the top 10 predicted potential PKIs with the NCI-60 GI50 profiles of compounds from the “marketed drugs” set. Table 6 shows the algorithm’s calculated correlation results for each of the top 10 potential PKIs.

The NCI’s COMPARE method revealed that 4 of the 10 compounds analyzed have a similar anti-proliferative profile with that of marketed PKIs. The lack of marketed PKIs drugs correlated with the other six compounds does not mean they don’t possess the capacity to inhibit PK, but rather that their profile is significantly different from that of all marketed anticancer drugs. These results offer an external validation of the proposed PKIs identification method.

The top ranking compound NSC 693255 and the 4th ranking NSC 669364 are close analogs and the results of NCI’s COMPARE methods indicate EGFR as potential target based on the antiproliferative profile similarity with erlotinib and gefitinib. All these compounds also share a clear chemical similarity highlighting the importance of the 4-(phenylamino)quinazoline scaffold (structure not shown).

## 3. Discussion

To summarize, for each compound, the upper outlier values represent cell lines that are more sensitive to a compound’s growth inhibitory activity than the others, meaning the respective compound acts by interfering with (at least) an essential mechanism for the proliferation of that specific cell line. Lower outliers, on the contrary, represent cell lines that are more resistant to a certain compound’s mechanism of action. Our method of identifying PKI compounds based on outliers, taking into account only the most sensitive cell lines, has the advantage of identifying potential drugs that are more selective than those found through a simple screening method. Upper outliers, especially, proved to be more significant in profiling a certain compound, as it is easily understandable that we are more interested in cell lines for which the respective compound has a higher growth inhibition activity.

AODs were used as a negative control group to reduce the probability of finding general-acting proliferation inhibitors and increase the method’s accuracy toward specifically selective inhibitors of cell proliferation.

The main purpose of the proposed method was to readily orient a medicinal chemist about potential structures or scaffolds to be used for PKI development, through either rational design or even drug repurposing. The method estimates the potential of an analyzed compound to be an inhibitor of one or more protein kinases, and should not be seen as a screening tool for PKI identification when used alone. Rather, the analysis could be corroborated with other known methods for PKI discovery, such as physicochemical descriptor filtering, scaffold hopping, etc. Yet, the indicated compounds resulting from the prediction should be further looked into, as the chemical space described by the resulting compounds appear to be larger than currently indicated by the state of the art knowledge in the field of protein kinase inhibition. Our method encompasses a profile of PKI inhibition as general as possible. The reasoning was that even if the signaling pathways are different, if two cell lines are sensitive to two different drugs acting through different mechanisms, by inhibiting different kinases, a third compound, potential PKI, to which both of the cell lines are sensitive to, may act at least through inhibiting one of the kinases.

Replacing missing datapoints with the mean value of the other results for a given compound was done in order for the Pearson correlation analysis and score calculations to be performed, as Pearson analysis doesn’t take into account compound lines with missing values. The replacing average values could represent false-negative results, as the results for some untested cells could have been upper or lower outliers, changing the inhibition profile of a compound. However, the pursued attribute in the performed analysis was the specificity of the outlier identification method, not sensitivity, therefore false-negative results would not cause an error as large as false-positives results would.

One advantage of this method would be that it does not require the exact chemical structure of the compound for the analysis, although a brief glimpse would help a medicinal chemist to get an idea about similar compounds, as a guide in which inhibition profiles should be compared. The growth inhibition profiles provided by the NCI can be analyzed without knowing specifics about the compounds—a useful trait regarding a large number of compounds that can be taken into account for analysis. Our method is simple and easily accessible because it is not necessary for chemical data-management software, as the calculations and statistics can be performed with only a spreadsheet application.

Another major upside of the proposed method is the speed of computation and the wide range of applicability, as it can be optimized for identifying ligands for different classes of targets involved in tumoral development, such as ion channels, enzymes, receptors, etc. The method can be also extended to other pharmacological domains, provided data related to the pharmacological activity is analyzed. Moreover, such a prediction could be narrowed to a singular molecular target, such as an isoform of an enzyme or a specific protein, as this would increase the probability of observing a specific pattern in the growth inhibition profile, which could better predict some potential ligands for the targeted molecule.

The limitations of the method are mainly related to data availability for creating the prediction set, as the accuracy of the scoring method would be higher if more PKIs were to be analyzed. Even with over 200 known inhibitors available in the PKIDB, only a fifth could be extracted and used for prediction, as the other PKIs lacked data, partially or even totally, in the GI50 NCI database.

We must also take into account the unpredictability of the pharmacokinetic behavior of some compounds, as two inhibitors may elicit similar responses in cell line testing, while in vivo it is possible for them to display different bioavailabilities at the site of action. The reverse of the medal is also applicable, as compounds with different growth inhibition test results on a cell line could present similar bioavailabilities, considering the multitude of reactions that a substance can undergo in situ. Catabolism of a molecule in development plays an important role in its pharmacological activity, especially when talking about entering tumoral cells, hence slight variations in the growth inhibition profile of a potential drug and that of a compound from the predictive set should not discourage the belief that the two substances may act similarly. Additionally, the static vs dynamic nature of the in vitro and in vivo models, as well as the mono culture and multi-cellular nature of the models can impact translational relevance.

## 4. Materials and Methods

### 4.1. Creation and Preparation of the Testing Dataset

Data regarding the growth inhibition profiles of the compounds were collected from the NCI database, (DTP NCI Bulk Data for Download GI50 data, June 2016 Release [39]) presenting compounds tested on the NCI-60 cell lines panel. Data were filtered for results expressed only as pGI50, calculated only from μM concentrations.

Descriptive analysis for the resulting lines was performed in order to identify statistical outliers. The statistical method used for outlier flagging was based on Tukey’s fences, using IQR (interquartile range) to define normally distributed values. Based on this method, outliers represent values not included in the following range:

[Q_1_ − 1.5*IQR, Q_3_ + 1.5 × IQR], (1)

where IQR = Q_3_ − Q_1_, and Q_1_ and Q_3_ represent first and third quartiles, respectively.

Therefore, upper outlier values were defined as a result of a cell line where the value of pGI50 is greater than the upper fence of Q_3_ + 1.5*IQR for that compound line, while lower outliers represent values lower than the lower fence of Q_1_ − 1.5 × IQR. Only lines of compounds presenting in the NCI database at least 50 results out of 60 totally possible were selected, in order to maintain a balance between having a large enough sample for analysis and avoiding insufficient data for each of these compounds.

### 4.2. Creation and Preparation of the Predictive Set

The predictive set was created similarly by selecting protein kinase inhibitors and approved oncologic drugs also tested on the NCI-60 panel and available in the NCI database. The PKI group was formed by interrogating the PKIDB (accessed September 2019) containing 218 known kinase inhibitors [7]. Its compounds have been searched for in the NCI database through chemical and numerical identifiers such as chemical structure, CAS (Chemical Abstracts Service) number, InChI (IUPAC International Chemical Identifier) key and NSC identifier. The resulting set was also filtered for compound lines displaying at least 50 datapoints out of 60.

The non-PKI group from the same set was created from already-approved drugs used for the treatment of different types of cancer, known to act through various pharmacological mechanisms except protein kinase inhibition (for example, alkylating agents, tubulin inhibitors or antimetabolites). The approved oncology drugs set VI (AOD6) dataset containing approved drugs extracted from the NCI database was downloaded from the NCI website [40]. Although most of them could be traced by the NSC identifier, several compounds proved to have too many missing datapoints to be taken into account for the study. The remaining drugs formed the AOD group (approved oncology drugs), the negative control of the predictive set. The negative control group was formed with oncologic drugs to make sure that growth inhibition through mechanisms unrelated to protein kinase inhibition was ruled out, as the selected AODs are known to be able to regulate cell growth through other mechanisms.

In order for the analysis to be possible, missing datapoints of the predictive set had to be replaced, therefore we chose to fill missing values with the average value of the other available results of that compound line for each row. This step was done in order to minimize the false-positive results, as the missing values would not be interpreted as outliers. However, statistical descriptors and outliers were calculated before replacing the missing values, as the added average values would modify some statistical parameters, such as quartile values and upper and lower fences, leading to the identification of a different number of outliers.

### 4.3. Establishing a Predictive Method

#### 4.3.1. Calculating the Weight Factor for Each Cell Line

The frequency of outliers in each group was used to calculate a weight factor for each cell line in order to be able to predict a score of PKI inhibition ability of other compounds. For each cell line, we calculated the frequency of upper and lower outlier appearances in the PKI set, and in the AOD set, respectively. The weight factors for each cell line were calculated using the difference between the frequencies in the PKI and AOD sets:

(2) $$WUi=\left(\frac{uPKI_{i}}{nPKI}-\frac{uAOD_{i}}{nAOD}\right)\times 100$$

(3) $$WLi=\left(\frac{lPKI_{i}}{nPKI}-\frac{lAOD_{i}}{nAOD}\right)\times 100$$

where WU_i_ represents the upper weight factor for cell line i, WL_i_ represents the lower weight factor for cell line i, uPKI_i_ and uAOD_i_ represent the number of upper outliers present in the PKI set, respectively in the AOD set, for cell line i, and similarly for lPKI_i_ and lAOD_i_ in regard to lower outliers. nPKI and nAOD represent the total number of compounds forming each of the sets. The weight factor is similar to the probability for a pGI50 value to be an outlier in the PKI set and not in the AOD set.

The quality of being an upper outlier was coded as a binary variable (U_i_), while the quality of being a lower outlier was coded as another binary variable (L_i_) for each cell line of the total of 60, as follows:

(4) $$U_{i}=\{\begin{array}{c} 1\;\mathrm{if}\;\mathrm{pGI}50\;\mathrm{value}\;\mathrm{for}\;\mathrm{cell}\;\mathrm{line}\;i\;\mathrm{represents}\;\mathrm{an}\;\mathrm{upper}\;\mathrm{outlier}\;\mathrm{value}\\ 0\;\mathrm{if}\;\mathrm{pGI}50\;\mathrm{value}\;\mathrm{for}\;\mathrm{cell}\;\mathrm{line}\;i\;\mathrm{is}\;\mathrm{not}\;\mathrm{an}\;\mathrm{upper}\;\mathrm{outlier}\;\mathrm{value} \end{array}$$

(5) $$L_{i}=\{\begin{array}{c} 1\;\mathrm{if}\;\mathrm{pGI}50\;\mathrm{value}\;\mathrm{for}\;\mathrm{cell}\;\mathrm{line}\;i\;\mathrm{represents}\;a\;\mathrm{lower}\;\mathrm{outlier}\;\mathrm{value}\\ 0\;\mathrm{if}\;\mathrm{pGI}50\;\mathrm{value}\;\mathrm{for}\;\mathrm{cell}\;\mathrm{line}\;i\;\mathrm{is}\;\mathrm{not}\;a\;\mathrm{lower}\;\mathrm{outlier}\;\mathrm{value} \end{array}$$

These variables were multiplied by cell line weights WU_i_ in the case of upper outliers, and WL_i_ for lower outliers, respectively. The sum of these two products for all 60 cell lines constituted a score (S_c_) for any given compound. Score calculation was done using the following formula:

(6) $$S_{c}=\overset{60}{\underset{i=1}{\sum }}(WU_{i}\times U_{i}+WL_{i}\times L_{i})$$

where c represents the identifier number for a compound, and i represents the NCI-60 cell line number, as presented in Table 2.

#### 4.3.2. Results Analysis and Validation

The scoring method was verified by ROC analysis using IBM SPSS Statistics 20 software and score results were correlated with the upper fence value (IQR + Q_3_) for each compound cell line to identify the most probable PKIs. Only compounds with score values larger than 10 that also presented an upper fence value for the compound’s results larger than 6 were chosen. The second filter was added in order to select compounds that elicited growth inhibition responses larger than the average value, for identifying significantly more potent inhibitors.

External validation of the results was performed using NCI’s COMPARE algorithm, by individually entering the NSC identifiers for each of the top 10 predicted potential PKIs. The algorithm calculated the correlation for each of the compounds with the NCI-60 GI50 profiles of other drugs (dataset chosen for comparison was “marketed drugs” and a minimum correlation coefficient of 0.4 was selected).

## 5. Conclusions

In summary, we have developed a statistical method based on outlier identification which analyses data from the growth inhibition profile tested on the NCI-60 panel in order to predict the potential of 9137 tested compounds of being a protein kinase inhibitor. The similarities and differences between the tested compounds and the profiles of 18 already-developed PKIs and 80 approved oncological drugs acting through non-PKI related mechanisms were appreciated using a scoring system based on statistical outlier distribution and pGI50 values, which indicated 409 compounds to have the best chance of being protein kinase inhibitors. Further analyses of these compounds need to be performed in order to determine their protein kinase inhibitor properties, possibly followed by in silico studies for confirmation. This method, while far from flawless, may appear to be a rapid and simple tool for aiding a medicinal chemist with basic knowledge of statistical analysis in choosing potential scaffolds or structures for further development as PKIs or other applicable targeted pharmacological classes.

## Figures and Tables

**Table 1:** Descriptive statistics for the testing and predictive sets.

Descriptives	Testing Set	Predictive Set	
		PKI Group	AOD Group
No. of compounds	9137	18	80
No. of cell lines	60	60	60
Total datapoints	548,220	1080	4800
Missing values	33,861	49	122
Average no. of datapoints/compound	56.29	57.28	58.47
Average pGI50 value	5.10	5.74	5.46
Total no. of outliers	36,570	69	253
No. of outliers/compound	1–22 (Avg* = 4.00)	0–8 (Avg = 3.83)	0–14 (Avg = 3.16)
Range*	0.002–6.65 (Avg = 1.58)	0.54–3.98 (Avg = 2.27)	0.12–4.4 (Avg = 1.98)
Standard deviation	0.0003–1.6617 (Avg = 0.3022)	0.1168–1.3622 (Avg = 0.5116)	0.028–0.9999 (Avg = 0.4242)

No. = number; Avg* = average value; Range* (for a compound) = difference between the maximum value and minimum value for all of the compound’s datapoints; PKI = protein kinase inhibitors; AOD = approved oncologic drugs; pGI50 = negative log values of 50% growth inhibition concentration.

**Table 2:** Cancer cell lines encompassed in the NCI-60 panel with their weights calculated based on lower and upper outlier frequency in each of the predictive set’s groups, sorted by represented tissue.

Cell No.	Cell Line	Tumoral Tissue Type	Cell line Weight Factors		Cell No.	Cell Line	Tumoral Tissue Type	Cell line Weight Factors	
			Upper Outliers	Lower Outliers				Upper Outliers	Lower Outliers
1	CCRF-CEM	Leukemia	−4.44	0.00	31	M14	Melanoma	5.56	−2.50
2	HL-60(TB)	Leukemia	−20.00	5.56	32	MDA-MB-435	Melanoma	3.06	0.00
3	K-562	Leukemia	11.67	11.11	33	SK-MEL-2	Melanoma	1.81	−1.25
4	MOLT-4	Leukemia	−6.94	−1.25	34	SK-MEL-28	Melanoma	11.11	−3.75
5	RPMI-8226	Leukemia	−1.94	5.56	35	SK-MEL-5	Melanoma	3.06	0.00
6	SR	Leukemia	−6.39	5.56	36	UACC-257	Melanoma	1.81	−2.50
7	A549/ATCC	NSCLC	−1.25	−1.25	37	UACC-62	Melanoma	1.81	−1.25
8	EKVX	NSCLC	27.78	−6.25	38	IGROV1	Ovarian	1.81	−1.25
9	HOP-62	NSCLC	0.00	4.31	39	OVCAR-3	Ovarian	0.00	3.06
10	HOP-92	NSCLC	8.61	−3.75	40	OVCAR-4	Ovarian	−1.25	−7.50
11	NCI-H226	NSCLC	11.11	4.31	41	OVCAR-5	Ovarian	5.56	−2.50
12	NCI-H23	NSCLC	−1.25	−1.25	42	OVCAR-8	Ovarian	0.00	−1.25
13	NCI-H322M	NSCLC	27.78	−5.00	43	NCI/ADR-RES	Ovarian	−1.25	−14.44
14	NCI-H460	NSCLC	−10.00	−1.25	44	SK-OV-3	Ovarian	9.86	9.86
15	NCI-H522	NSCLC	1.81	0.00	45	786-0	Renal	−3.75	4.31
16	COLO 205	Colon	5.56	−1.25	46	A498	Renal	24.03	4.31
17	HCC-2998	Colon	−2.50	−1.25	47	ACHN	Renal	15.42	−5.00
18	HCT-116	Colon	4.31	4.31	48	CAKI-1	Renal	17.22	−7.50
19	HCT-15	Colon	−2.50	−7.50	49	RXF 393	Renal	4.31	0.00
20	HT29	Colon	9.86	0.00	50	SN12C	Renal	0.00	−1.25
21	KM12	Colon	16.67	4.31	51	TK-10	Renal	16.67	−6.25
22	SW-620	Colon	0.00	5.56	52	UO-31	Renal	3.06	−8.75
23	SF-268	CNS	0.00	0.00	53	PC-3	Prostate	−1.25	−2.50
24	SF-295	CNS	−1.25	0.00	54	DU-145	Prostate	−2.50	−1.25
25	SF-539	CNS	4.31	5.56	55	MCF7	Breast	−2.50	0.00
26	SNB-19	CNS	0.00	−0.69	56	MDA-MB-231/ATCC	Breast	−1.25	−5.00
27	SNB-75	CNS	6.11	−1.25	57	MDA-MB-468	Breast	−3.75	0.00
28	U251	CNS	−1.25	5.56	58	HS 578T	Breast	7.36	−2.50
29	LOX IMVI	Melanoma	−3.75	0.00	59	BT-549	Breast	1.81	−3.75
30	MALME-3M	Melanoma	5.56	−1.25	60	T-47D	Breast	−3.75	1.81

NSCLC = non-small cell lung cancer; CNS = central nervous system; NCI-60 = National Cancer Institute’s previously tested compounds on 60 tumoral cell lines.

**Table 3:** Descriptive statistics for the 409 compounds identified as potential PKIs.

Descriptives	Resulted Compounds Set
Number of compounds	409
Total datapoints	24,540
Missing values	2207
Average pGI50	5.90
Total outliers	1907
No. outliers/compound	1–19 (Avg = 4.66)
Range	0.44–6.66 (Avg = 2.03)
Standard deviation	0.0914–7.1358 (Avg = 0.4363)
Score values	10–118.33 (Avg = 19.66)
SD of score values	10.1343
Upper fence values	5.02–10.19 (Avg = 6.61)
SD of upper fence values	0.7577

Upper fence-SD = standard deviation, Avg* = average value.

**Table 4:** Descriptive statistics for the computed physicochemical properties of the 409 predicted potential PKIs.

	Minimum	Maximum	Mean	Standard Deviation
Molecular weight	119.19	1546.61	423.55	186.92
cLogP	−13.21	13.71	3.25	2.90
No. of H-Acceptors	0	45	5.78	4.82
No. of H-Donors	0	28	1.51	2.62
Total surface area	91.17	1084.60	295.57	134.93
Relative polar surface area	−0.01	0.69	0.23	0.12
Molecular flexibility	0	0.86	0.37	0.18
Molecular complexity	0.38	1.28	0.83	0.14
No. of non-C/H atoms	1	47	6.88	4.93
No. rotatable bonds	0	40	5.89	6.12
No. rings closures	0	20	3.40	2.00
No. of aromatic rings	0	8	2.06	1.49

**Table 5:** Top 10 best scoring compounds.

Compound	Score	Upper Fence	Upper Outlier Count	Outlier Cell Lines
NSC 693255	118.33	6.278	8	ACHN, CAKI-1, EKVX, IGROV1, NCI-H322M, NCI-H522, SK-OV-3, TK-10
NSC 686288	62.5	6.435	11	A498, CAKI-1, IGROV1, K-562, MCF7, NCI-H460, OVCAR-5, SW-620, T-47D, TK-10, UACC-257
NSC 665910	54.16	7.016	2	A549/ATCC, HS 578T, NCI-H226, NCI-H322M, SF-295, SK-OV-3, SNB-19, TK-10
NSC 669364	52.36	6.225	5	ACHN, DU-145, EKVX, NCI-H522, SK-OV-3
NSC 24112	52.08	6.120	6	A498, HCT-116, HOP-92, HT29, K-562, SR
NSC 22323	51.94	7.234	9	CCRF-CEM, EKVX, HCT-116, HOP-62, HOP-92, HT29, K-562, MOLT-4, NCI-H23, RPMI-8226
NSC 61805	49.30	6.276	6	COLO 205, EKVX, MCF7, MOLT-4, PC-3, RXF 393, TK-10
NSC 239072	48.75	8.003	13	A498, A549/ATCC, ACHN, COLO 205, HCC-2998, HL-60(TB), HOP-92, K-562, MDA-MB-231/ATCC, MDA-MB-435, NCI-H460, SF-295, TK-10
NSC 676469	48.61	6.093	6	ACHN, HCT-15, K-562, M14, TK-10, UACC-62
NSC 650395	47.77	8.784	9	A498, BT-549, CAKI-1, HCC-2998, HOP-62, HOP-92, MCF7, MDA-MB-231/ATCC, OVCAR-3, PC-3, RXF 393, SF-295, SK-OV-3, SNB-19, UACC-62

Upper outlier = any pGI50 value bigger than the upper fence (1,5 * IQR); IQR = interquartile range; NSC = Cancer Chemotherapy National Service Center number.

**Table 6:** Top 10 predicted potential PKIs correlated with known PKI drugs using COMPARE algorithm.

Compound	Correlation Found with at Least One Compound (Pearson>0.4)	Correlation with PKI (with Correlation Score)
NSC 693255	Yes	erlotinib (0.76) lapatinib (0.71) gefitinib (0.71) dasatinib (0.43)
NSC 686288	No	-
NSC 665910	No	-
NSC 669364	Yes	erlotinib (0.63) gefitinib (0.50) lapatinib (0.48) dasatinib (0.41)
NSC 24112	Yes	imatinib (0.44)
NSC 22323	Yes	imatinib (0.44)
NSC 61805	No	-
NSC 239072	No	-
NSC 676469	No	-
NSC 650395	No	-

## Supplementary Materials

The following are available online at https://www.mdpi.com/1420-3049/25/8/1766/s1, Table S1: Detailed descriptive statistics for the PKI group of the predictive set, Table S2: Summary of descriptive statistics for the testing set compounds (n = 9137), Table S3: ROC analysis of score prediction accuracy, tested on the predictive set (PKI vs. AOD), Table S4: Summary of descriptive statistics for the 409 potential PKI compounds.

## Abbreviations

AOD	approved oncology drugs
CAS	Chemical Abstracts Service registry number
CNS	central nervous system
DTP	Developmental Therapeutics Program
GI50	half maximal growth inhibition
IC50	half maximal inhibitory concentration
InChI	IUPAC International Chemical Identifier
IQR	interquartile range
LC50	half maximal lethal concentration
NCI	National Cancer Institute
NSC	Cancer Chemotherapy National Service Center number
NSCLC	non-small cell lung cancer
PKI	Protein Kinase Inhibitor
ROC	receiver operating characteristic
TGI	total growth inhibition
