# Supplementary material for: In Search of Outliers. Mining for Protein Kinase Inhibitors Based on Their Anti-Proliferative NCI-60 Cell Lines Profile
Source: Molecules. 2020 Apr 11;25(8):1766. doi: 10.3390/molecules25081766 (PMC7221881; doi:10.3390/molecules25081766)
Supplement: Supplementary file 1 [file molecules-25-01766-s001.pdf]

molecules

Supplementary Materials

In search of outliers. Mining for protein kinase inhibitors based on their anti-proliferative NCI-60 cell lines profile

George Nicolae Daniel Ion [1] and George Mihai Nitulescu [1,*]

[1] Faculty of Pharmacy, "Carol Davila" University of Medicine and Pharmacy, Traian Vuia 6, Bucharest, 020956, Romania; E-Mail: daniel.ion@drd.umfcd.ro (G.N.D.I.); nitulescu_mihai@yahoo.com (G.M.N.);
* Correspondence: nitulescu_mihai@yahoo.com; george.nitulescu@umfcd.ro (G.M.N.)

Table S1. Detailed descriptive statistics for the PKI group of the predictive set

NSC no.	Missing	Up outl.	Low outl.	Min	Max	Mean	Range	SD	Q1	Q3	IQR	Upper fence	Lower fence
701554	9	2	2	4.06	5.31	4.73	1.25	0.2874	4.60	4.87	0.27	5.28	4.19
702827	5	3	4	4.23	5.36	4.53	1.13	0.1584	4.47	4.56	0.10	4.71	4.32
715055	0	8	0	4.77	7.35	5.45	2.58	0.6202	5.06	5.53	0.48	6.25	4.34
718781	1	2	0	4.01	7.01	5.14	3.00	0.7358	4.59	5.44	0.85	6.71	3.31
732517	0	0	0	4.91	8.35	6.42	3.44	1.1145	5.32	7.51	2.19	10.79	2.04
741078	3	0	0	4.02	8.00	5.57	3.98	1.3152	4.47	6.24	1.78	8.91	1.80
743414	1	6	0	4.41	7.63	4.82	3.21	0.4544	4.65	4.82	0.17	5.07	4.40
745750	0	4	0	4.52	7.44	5.49	2.92	0.6100	5.05	5.62	0.57	6.47	4.20
747599	1	1	2	4.79	8.00	5.56	3.21	0.4388	5.38	5.72	0.34	6.23	4.86
747971	0	0	0	5.36	5.90	5.63	0.54	0.1158	5.54	5.73	0.18	6.00	5.27
750690	0	2	1	4.80	7.25	5.63	2.45	0.3267	5.48	5.78	0.30	6.24	5.02
750691	1	8	0	5.31	8.00	6.09	2.69	0.6745	5.67	6.10	0.43	6.73	5.03
751249	0	0	4	7.15	8.00	7.89	0.85	0.1741	7.82	8.00	0.18	8.27	7.54
752782	9	7	4	4.34	6.43	5.43	2.09	0.2659	5.35	5.48	0.13	5.68	5.15
757441	2	5	0	4.26	6.49	5.11	2.23	0.4773	4.85	5.30	0.45	5.98	4.17
760766	2	8	1	5.10	6.56	5.78	1.46	0.3405	5.56	5.85	0.30	6.30	5.11
761431	0	8	0	5.06	7.83	5.72	2.76	0.7061	5.31	5.68	0.37	6.24	4.75
683246	1	2	2	4.24	5.34	4.74	1.10	0.1971	4.63	4.83	0.20	5.12	4.33

Outl. = outliers; SD = standard deviation; Q1, Q3 = quartiles; IQR = interquartile range (Q3-Q1).

Table S2. Summary of descriptive statistics for the testing set compounds (n = 9137)

Descriptives	Mean	Standard Error	Median	Standard Deviation	Range	Minimum	Maximum	Sum	Confidence Level(95,0%)
Number of datapoints	56.29	0.03	57	2.8393	10	50	60	514359	0.0582
Missing	3.71	0.03	3	2.8393	10	0	10	33861	0.0582
1st quartile	4.95	0.01	4.7155	0.9568	9.6	1	10.6	45244.07	0.0196
3rd quartile	5.22	0.01	4.902	1.0651	10	1	11	47679.48	0.0218
IQR	0.27	0	0.231	0.2177	1.653	0	1.653	2435.41	0.0045
Upper fence	5.62	0.01	5.2775	1.2809	11.4795	1	12.4795	51332.6	0.0263
Lower fence	4.55	0.01	4.3395	0.8745	9.6	1	10.6	41590.95	0.0179
Upper outliers	2.68	0.03	2	2.771	14	0	14	24475	0.0568
Lower outliers	1.32	0.02	0	2.1263	15	0	15	12095	0.0436
Total outliers	4	0.03	3	3.1582	21	1	22	36570	0.0648
Range	1.58	0.01	1.414	0.9272	6.655	0.002	6.657	14471.86	0.019

Table S3. ROC analysis of score prediction accuracy, tested on the predictive set (PKI vs. AOD)

Positive if Greater Than or Equal To	Sensitivity	1 - Specificity	Positive if Greater Than or Equal To	Sensitivity	1 - Specificity
-63.64	1	1.00	-6.32	1.00	0.50
-58.61	1	0.99	-5.35	1.00	0.49
-54.51	1	0.98	-4.10	1.00	0.48
-51.74	1	0.96	-3.13	1.00	0.45
-47.50	1	0.95	-2.22	1.00	0.43
-45.63	1	0.94	-1.74	1.00	0.41
-44.31	1	0.93	-1.11	1.00	0.40
-40.76	1	0.91	-0.56	1.00	0.39
-37.99	1	0.90	-0.21	1.00	0.38
-37.43	1	0.89	0.91	0.83	0.13
-36.39	1	0.88	2.64	0.83	0.11
-35.14	1	0.86	3.54	0.83	0.10
-32.64	1	0.85	3.96	0.83	0.09
-30.42	1	0.84	5.14	0.83	0.08
-28.27	1	0.83	7.29	0.83	0.06
-26.04	1	0.81	10.21	0.83	0.05
-25.14	1	0.79	14.66	0.78	0.05
-22.99	1	0.78	17.57	0.78	0.04
-21.04	1	0.76	18.27	0.78	0.03
-20.35	1	0.75	20.21	0.78	0.01
-19.86	1	0.74	21.74	0.72	0.01
-19.24	1	0.73	22.99	0.67	0.01
-18.27	1	0.71	25.28	0.61	0.01
-17.71	1	0.70	26.81	0.56	0.01
-17.57	1	0.69	32.29	0.50	0.01
-17.22	1	0.68	37.78	0.50	0.00
-15.69	1	0.66	41.19	0.44	0.00
-13.19	1	0.63	49.94	0.39	0.00
-11.67	1	0.61	57.30	0.33	0.00

-11.11	1	0.60	63.20	0.28	0.00
-10.35	1	0.59	74.72	0.22	0.00
-9.59	1	0.58	108.20	0.17	0.00
-9.17	1	0.56	137.44	0.11	0.00
-8.27	1	0.55	141.19	0.06	0.00
-7.02	1	0.54	142.81	0.00	0.00
-6.46	1	0.53			

Table S4. Summary of descriptive statistics for the 409 potential PKI compounds

	Mean	Standard Error	Median	Standard Deviation	Range	Min	Max	Sum	Confidence Level(95,0%)
Count cells	54.47	0.52	59.00	10.5398	56.00	5.00	61.00	22277.57	1.02
1st quartile	5.65	0.04	5.54	0.8698	5.20	4.04	9.25	2309.94	0.08
3rd quartile	6.02	0.04	5.80	0.8295	9.21	0.36	9.57	2461.98	0.08
IQR	0.40	0.02	0.33	0.3719	6.47	0.00	6.47	164.07	0.04
Upper fence	6.61	0.04	6.33	0.7577	5.17	5.03	10.20	2703.04	0.07
Lower fence	5.06	0.05	5.10	1.0800	6.47	2.53	9.00	2070.61	0.10
Upper outliers	2.97	0.12	2.00	2.4221	13.00	0.00	13.00	1215.00	0.24
Lower outliers	1.69	0.11	1.00	2.2607	14.00	0.00	14.00	692.00	0.22
Total outliers	4.66	0.16	4.00	3.1962	18.00	1.00	19.00	1905.00	0.31
Range	2.03	0.04	1.85	0.8227	6.22	0.44	6.66	828.91	0.08
Score	19.67	0.50	16.67	10.1343	108.33	10.00	118.33	8043.06	0.99

© 2020 by the authors. Submitted for possible open access publication under the terms and conditions of the Creative Commons Attribution (CC BY) license (http://creativecommons.org/licenses/by/4.0/).
